# Altered reward processing following an acute social stressor in adolescents

**DOI:** 10.1371/journal.pone.0209361

**Published:** 2019-01-04

**Authors:** Sarah Hope Lincoln, Angela Pisoni, Erin Bondy, Poornima Kumar, Paris Singleton, Greg Hajcak, Diego A. Pizzagalli, Randy P. Auerbach

**Affiliations:** 1 Department of Psychiatry, Harvard Medical School, Boston, MA, United States of America; 2 Center for Depression, Anxiety and Stress Research, Belmont, MA, United States of America; 3 Department of Psychology and Neuroscience, Duke University, Durham, NC, United States of America; 4 Department of Psychological and Brain Sciences, Washington University in St. Louis, St. Louis, MO, United States of America; 5 Departments of Biomedical Sciences and Psychology, Florida State University, Tallahassee, FL, United States of America; 6 McLean Imaging Center, Belmont, MA, United States of America; 7 Department of Psychiatry, College of Physicians and Surgeons, Columbia University, New York, NY, United States of America; 8 Division of Clinical Developmental Neuroscience, Sackler Institute, New York, NY, United States of America; The University of Melbourne, AUSTRALIA

## Abstract

Altered reward processing is a transdiagnostic factor implicated in a wide range of psychiatric disorders. While prior animal and adult research has shown that stress contributes to reward dysfunction, less is known about how stress impacts reward processing in youth. Towards addressing this gap, the present study probed neural activation associated with reward processing following an acute stressor. Healthy adolescents (*n* = 40) completed a clinical assessment, and fMRI data were acquired while participants completed a monetary guessing task under a *no-stress condition* and then under a *stress* condition. Based on prior literature, analyses focused on a priori defined regions-of-interest, specifically the striatum (win trials) and dorsal anterior cingulate cortex [dACC] and insula (loss trials). Two main findings emerged. First, reward-related neural activation (i.e., striatum) was blunted in the stress relative to the no-stress condition. Second, the stress condition also contributed to blunted neural response following reward in loss-related regions (i.e., dACC, anterior insula); however, there were no changes in loss sensitivity. These results highlight the importance of conceptualizing neural vulnerability within the presence of stress, as this may clarify risk for mental disorders during a critical period of development.

## Introduction

Adolescence is characterized by heightened reward sensitivity [1], increased stress exposure [2], and high rates of mental disorders [3]. Aberrant reward processing is implicated in a range of mental disorders including major depressive disorder [4, 5], post-traumatic stress disorder [6], and schizophrenia [7], and prior research also has demonstrated that stress robustly predicts the onset and severity of these disorders [8]. Building on these findings, recent research has sought to clarify how stress may impact reward functioning to improve our understanding of disorder etiology [9–11].

In animal studies testing the impact of physical stress (e.g., foot shock), results showed that rats exhibited reduced consummatory (i.e., decreased saccharine consumption; [12] and exploratory [13, 14] behaviors. Similarly, acute social stress (e.g., social defeat) led to a decrease in reward seeking behavior in adolescent rats [15]. In humans, early life adversity has been associated with reward dysfunction, as evidenced by blunted activation during both reward anticipation in the dorsal striatum [16, 17] and reward receipt in the ventral striatum [18–20]. The impact of chronic stress through active military service also contributed to reduced nucleus accumbens (i.e., ventral striatum) activation during reward receipt [21]. Using a physical stressor (cold pressor), Porcelli and colleagues (2012) demonstrated that acute stress decreased activation to monetary reward in the dorsal striatum and orbitofrontal cortex among healthy adults [22]. Collectively, animal and human studies show an association between stress and blunted behavioral and neural response to reward anticipation and consumption. However, these studies operationalize reward dysfunction following stress without considering baseline levels of reward processing. Probing *changes* in reward processing—by examining patterns of reward function before and after acute stress—may afford new insights into the development of mental disorders.

Towards addressing this gap, Kumar and colleagues administered a monetary incentive delay task to healthy adults while acquiring functional magnetic resonance imaging (fMRI) data under no-stress and stress conditions [23]. After the initial task administration, participants received acute negative feedback and then, completed the task a second time. Results showed reduced activation in the putamen and caudate during reward receipt following stress relative to no-stress, suggesting that stress can elicit anhedonic-like activation patterns. Although this study expands past research, little is known about how stress may elicit *changes* in reward function among adolescents. This downward extension is particularly important, as adolescence is a sensitive period for identifying emerging individual differences in reward functioning. Namely, adolescence marks a period of substantial changes in incentive-seeking behavior, characterized by a greater drive to approach pleasant experiences and potentially greater engagement in risk-taking behaviors [24]. Moreover, adolescence also constitutes a period of greater reliance on peer relationships [25] and greater sensitivity to peer rejection [26]. Taken together, understanding changes in reward processing as a function of social stressors may clarify potential vulnerability to the emergence of psychiatric symptoms.

The present study tested whether acute social stress negatively impacted reward-related neural functioning in healthy adolescents. Participants completed a monetary reward task [27, 28] under no-stress and stress conditions. Additionally, we implemented an ecologically valid social task [29, 30] in which participants believed they were being accepted or rejected by peers. This task was used as an acute social stressor, as participants were informed they were rejected more than other teens participating in the study. Consistent with prior research probing baseline reward function, (e.g., [31, 32]) we hypothesized that following acute social stress, adolescents would exhibit *reduced* activation in regions that have been found to respond to win feedback (e.g., striatum). Additionally, previous research has shown that loss feedback is associated with dACC and anterior insula activation [33–35], and therefore, we hypothesized that adolescents would exhibit *increased* activation in loss-related regions following stress.

## Materials and method

### Participants

The original sample included 61 adolescents from the greater Boston area. Data from 21 adolescents were excluded due to: (a) head movement (> 2 mm) or artifacts (*n* = 13), (b) < 10% change in affect following stress (*n* = 4), or (c) scanner malfunction (*n* = 4). The final sample included 40 adolescents (75% female) aged 12–14 years (*M* = 13.20, SD = 0.72; Tanner Stage: *M* = 3.22, *SD* = 0.48). The ethnic distribution was: 82.5% Caucasian, 7.5% Asian, 7.5% multiracial, and 2.5% Black or African American. Participants were right-handed, native English speakers, with normal or corrected to normal vision. Exclusion criteria included lifetime mental disorders, loss of consciousness (> 5 minutes), a neurological disease, or MRI contraindication. Excluded participants did not differ from the final sample on sex, or ethnicity (*p*s > .14). Relative to the final sample, excluded participants were younger (*t*(60) *= -*2.87, *p =* .006).

### Procedure

The Partners Institutional Review Board approved study procedures. Adolescents assented and legal guardians provided written consent in accordance with the Declaration of Helsinki. The baseline assessment included two study visits, occurring within 1–2 weeks of each other (*M* = 7.30 days, SD = 3.48). During the initial study visit, participants completed a clinical interview and self-report assessments of pubertal status. Adolescents also completed the first part of the Chatroom Task. Eligible participants completed a second visit in which fMRI data were acquired while completing the Guessing Task. In the scanner, participants completed one run of the Guessing Task (no-stress condition), followed by the Chatroom Task (the social stressor), and then a second administration of the Guessing Task (stress condition).

### Clinical instruments

The Kiddie-Schedule for Affective Disorders and Schizophrenia-Present (K-SADS-PL; [36]) is a semi-structured interview assessing lifetime DSM-IV disorders in youth. Interviews were recorded, and 20% of interviews were randomly selected to assess inter-rater reliability (κ = 1.00).

The Tanner Staging Questionnaire (TSQ; [37] is a 5-item self-report assessment of physical development.

### Experimental task

The Guessing Task [27] is designed to assess neural response to win and loss feedback and has been shown to reliably activate reward- and loss-related regions. For each trial, there was a jittered intertrial interval, which showed a fixation cross for 1300–9100 ms. Then, participants were presented with two identical doors and were instructed to select which door (left or right) they thought contained a prize as quickly as possible by pressing the left or right button on the response box, respectively, (doors presented up to 3900 ms). After the selection, a jittered fixation cross appeared for 1300–7800 ms, and then, feedback was presented for 1300 ms: (a) green “↑” for win or (b) red “↓” for loss. On each trial, the participant either won $0.50 or lost $0.25, and the participant was informed that they would keep the sum earned from the experiment. Unbeknownst to the participant, the outcome was fixed, as they won 24 trials and lost 24 trials in pseudorandom order, completing a total of 48 trials. For the current study, participants completed the Guessing Task under both *no stress* and *stress* conditions.

### Stress manipulation

The Chatroom Task [29, 30] was designed to simulate adolescent social interactions and to probe differential response to peer feedback (i.e., acceptance versus rejection). E-Prime (Psychological Software Tools, Pittsburgh, PA) software was used to present stimuli and record responses. In Phase 1, participants were led to believe they were participating in a study of how adolescents interact in online chatrooms. First, they created an online profile (i.e., indicating likes, dislikes) and then, accompanying photographs of the participants were taken. Next, they viewed 60 photographs of same-aged adolescents and selected 30 adolescents they were *interested* and 30 they were *not interested* in chatting with online following a neuroimaging scan 1–2 weeks later. Participants were informed that peers from collaborating institutions would review their profiles and indicate whether they were *interested* (i.e., peer acceptance) or *not interested* (i.e., peer rejection) in chatting online with them. Of note, female participants were only presented with female peers to select, and similarly, males could only select male peers.

For Phase 2, participants received peer feedback from the 60 adolescents allegedly participating in the nationwide study while fMRI data were acquired. During each trial, the participant viewed the photograph of a “participating adolescent” (1300 ms), and a photograph caption displaying *interested* or *not interested* was used to remind the participant about the prior selection. Then, a jittered fixation cross (1300–7600 ms) was presented, followed by the peer feedback under the photograph (2600 ms). After the feedback, a jittered fixation cross (1300–5200 ms) was displayed, and the participant received a prompt, “*How does this make you feel*?” and was instructed to provide a rating on a visual analogue scale ranging from 0 (*very bad*) to 100 (*very good*). Feedback was provided in pseudorandom order with no more than 3 trials of the same response provided consecutively. Unbeknownst to participants, feedback was fixed, as everyone received the same number of acceptance (30 *interested* trials) and rejection (30 *not interested* trials) trials.

**Fig 1:**
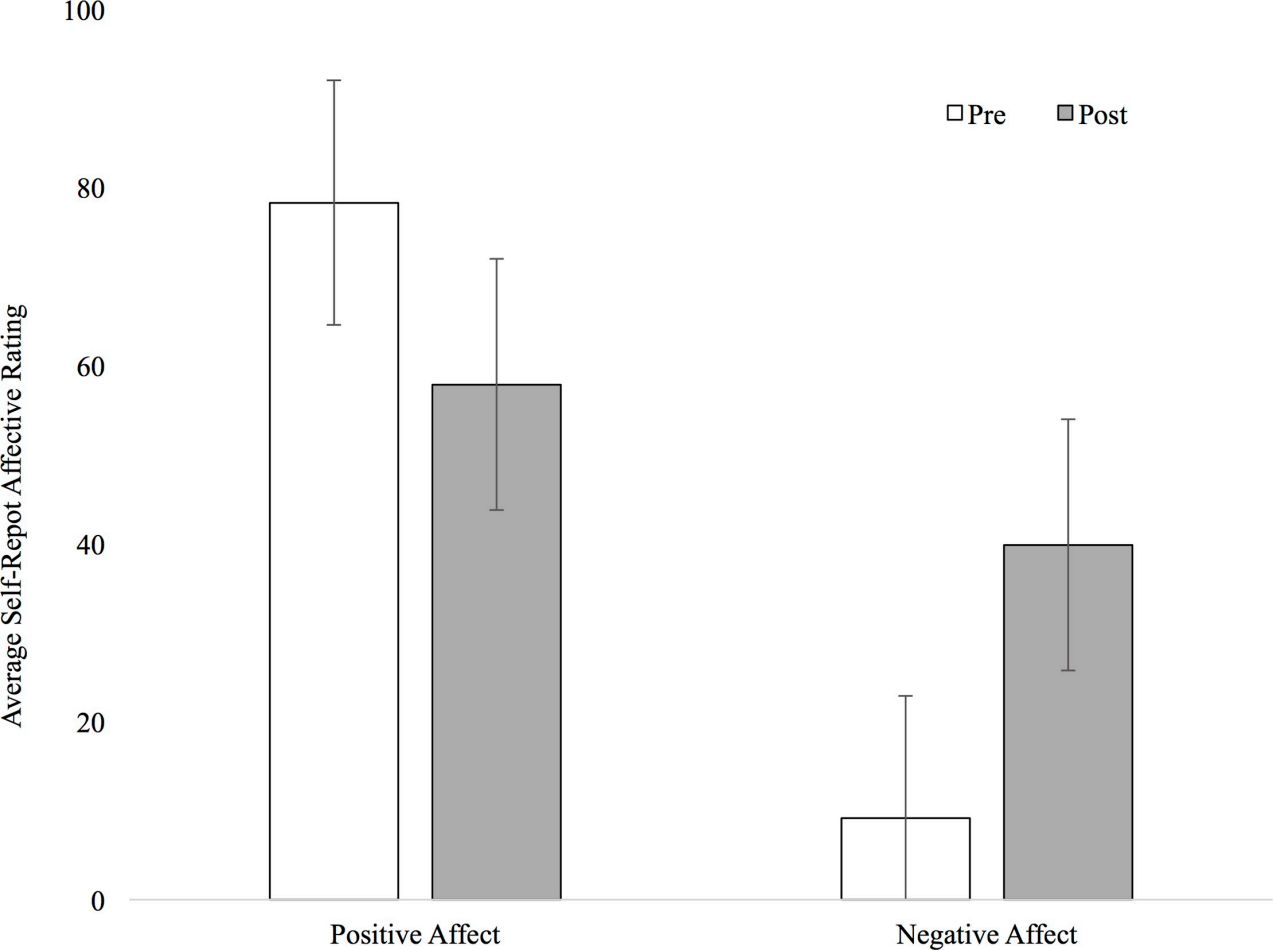
Self-reported negative and positive affect ratings *pre-stress* condition and *post-stress* condition.

The stressor included two components. First, at the completion of the Chatroom Task, the screen displayed the following non-veridical feedback, “**Individual Performance:** Peer Acceptance (38%), Peer Rejection: 62%; **Average Participant Performance:** Peer Acceptance: 64%, Peer Rejection: 36%.” In addition to reading this statement aloud through the scanner intercom, study staff stated the following, “*Based on the breakdown from today*, *it seems like you’re accepted by fewer teens compared to other teens completing the task*. *Additionally*, *you are being rejected more than other teens that have completed the selection process*.” Second, to provide a rationale as to why it was necessary to repeat the Guessing Task, study staff also read the following statement to participants, “*Unfortunately*, *your performance in the Guessing Task was below average*. *Remember*, *you earned only $12 out of a possible $24*. *For the data to be usable*, *a participant needs to earn more than $14*. *Thus*, *we’re going to need to redo this task*. *Please try to focus*.” To determine whether the stress manipulation was effective, participants completed visual analogue scales probing affect ratings prior to entering the scanner and following the two-pronged stressor. Participants rated positive (i.e., happy, joyful) and negative (i.e., sad, upset, and discouraged) items from the Positive and Negative Symptom Schedule (PANAS; [38]) using a sliding scale ranging from 0 (*not very true of me*) to 100 (*very true of me*). To determine whether the stress manipulation was effective a participant needed to exhibit a 10% increase in negative affect or a 10% decrease in positive affect from pre- to post-stress; participants who did not exhibit changes in negative or positive affect were excluded (*n* = 4). On average, participants’ negative affect increased 30.6% and positive affect decreased 19.8% following the stressor (Table 1) (Fig 1). In a repeated measures ANOVA, a *Time* x *Affect* interaction emerged *F*(1,39) = 138.05, *p* = 2.20 x 10^−14^, *η*^*2*^ = 0.78. Results show that following stress, positive affect significantly decreased (*p* = 3.29 x10^-10^, *η*^*2*^ = 0.89) and negative affect significantly increased (*p* = 2.99 x 10^−13^, *η*^*2*^ = 0.23). Imaging data from this social stressor task will be reported in future manuscripts.

**Table 1:** Positive and negative affect before and after stress (*n* = 40).

	No-Stress	Stress	*F*	*p*
	*M (SD)*	*M (SD)*		
Positive Affect	78.26 (14.13)	57.94 (16.04)	138.05	2.20 x 10^−14^
Negative Affect	9.14 (13.73)	39.79 (16.51)		

### Image acquisition and processing

Functional MRI data were acquired on a Siemens Tim Trio 3.0 Tesla MR scanner using a 32-channel head coil at the McLean Imaging Center. For functional scans, data were acquired in an interleaved fashion using T2*-weighted gradient echo planer images (EPI), with the following parameters TR/TE: 1300/32.2ms; FOV: 212 mm; phase partial Fourier 6/8; echo spacing = 0.69ms; matrix: 64x64; 72 slices; in-plane resolution: 2mm; flip angle = 66°; voxels 2 x 2 x 2 mm. Data were preprocessed and analyzed using SPM12 (Statistical Parametric Mapping [SPM]; http://fil.ion.u-cl.ac.uk/spm/). Preprocessing steps included: distortion and motion correction, slice timing, realignment to the mean image, coregistration to the structural image, normalization to the MNI template, and smoothing with a 4mm Gaussian kernel. The Artifact Detection Toolbox (ART; http://www.nitrc.org/projects/artifact_detect/) was used to identify outlier scans in global signal (> ±2 *SD*) and movement (> 2.5 mm of movement or 2 degrees of rotation from the previous volume. Participants for whom more than 15% of the 491volumes were removed due to movement, in either the pre-stress or post-stress run of the monetary reward task, were excluded (*n* = 13) [39]. General linear models were defined at the single-subject level with regressors corresponding to anticipation cue, decision cue, win feedback, and loss feedback trials. Contrast maps were constructed for win feedback > fixation and loss feedback > fixation. In order to reduce the influence of outliers, we chose to winsorize outlier data for each ROI; data were winsorized at 2.5 standard deviations from the mean. Approximately 2% of the data for the ROIs were winsorized.

**Fig 2:**
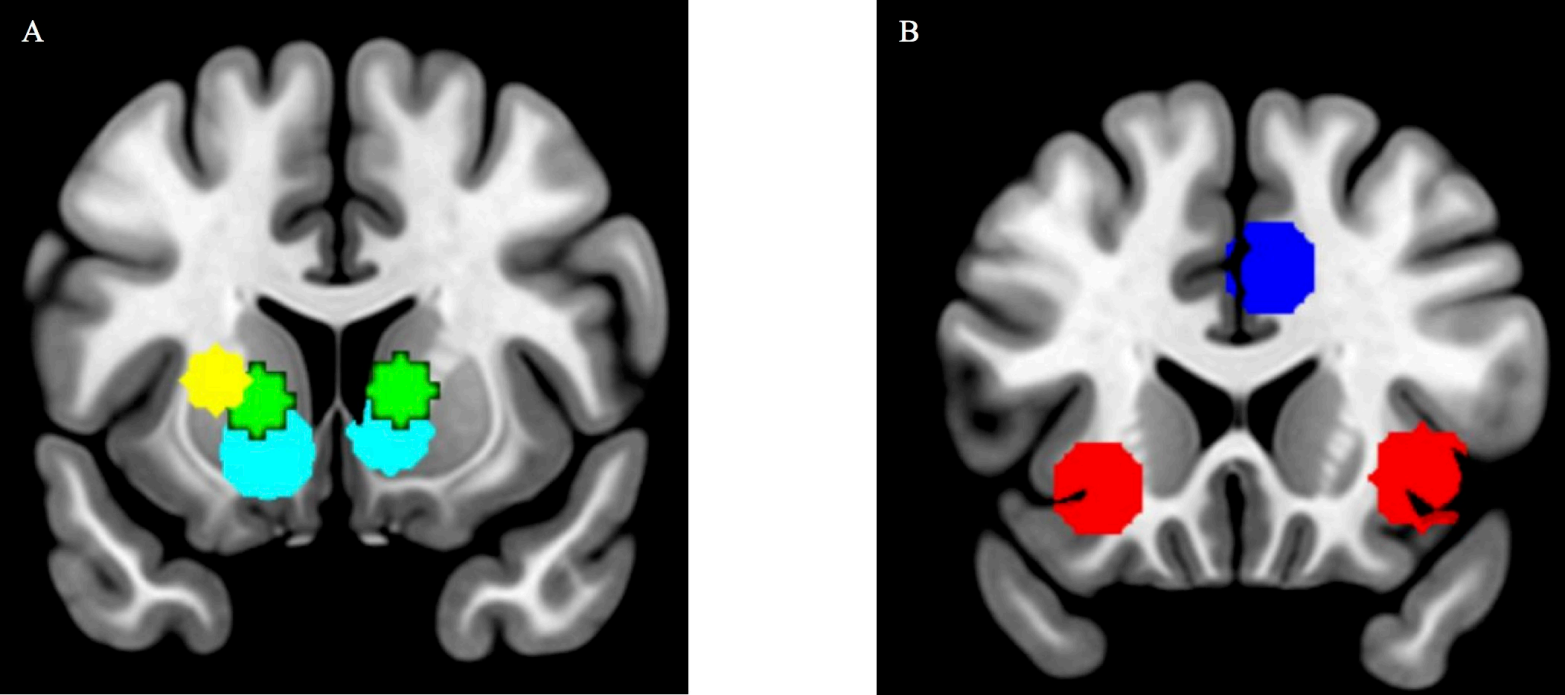
(A) Neuroanatomical regions of interest for left and right caudate (green), left putamen (yellow), and left and right ventral striatum (cyan), (B) Neuroanatomical regions of interest for dACC (blue) and left and right anterior insula (red).

To test *a priori* hypotheses that neural response to wins would decrease after stress, five 10-mm spherical regions of interest (ROIs) were created around MNI coordinates in regions previously implicated in reward processing. There are several methods by which ROIs are defined, including both anatomical and functional definitions. Anatomically defined ROIs have two significant challenges. First the specified regions may be much larger than the actual area of activation by the task. Second anatomically defined ROIs assume functional homogeneity across the region, which in many cases is an erroneous assumption [40]. These concerns would be particularly problematic with regions like the dACC and the insula included in our analyses. In order to avoid these problems, we used functional ROIs identified from prior studies of reward related regions [27, 41]. Within the ventral striatum, the left (x = -14, y = 10, z = -8) and right (x = 10, y = 6, and z = -4) ROIs were obtained from a prior publication using the same task [27]. In the dorsal striatum, coordinates for the left (x = -16, y = 20, z = 2) and right caudate (x = 12, y = 20, z = 4) were obtained [27]. The putamen did not emerge in Carlson et al., (2011), but was a region of interest in this study due to its role in stress and reward processing [32]. Thus, coordinates for the left putamen were obtained from a meta-analysis of reward related-neural networks in adults [41] and adolescents [32]. The right putamen did not emerge in the meta-analysis as a brain area commonly activated in reward-related processing, and thus was not used as an ROI in the present study. Using MarsBar [42] the left putamen ROI was defined as a 10-mm radius sphere centered at (x = -24, y = 4, z = 6) [41] (Fig 2A). There is approximately a 24 voxel overlap between the right ventral striatum and the right caudate, and a 54 voxel overlap between the left ventral striatum and the left caudate. No other ROIs overlap.

To probe regions associated with loss and rejection, the dorsal anterior cingulate cortex (dACC) and anterior insula ROIs were created from the same meta-analysis of regions implicated in neural networks associated with loss [41]. The dACC was defined as a 10-mm radius sphere centered at (x = 6, y = 24, z = 34). The left anterior insula was defined as a 10-mm radius sphere centered at (x = -28, y = 24, z = -8), and the right anterior insula as a 10-mm radius sphere centered at (x = 36, y = 20, z = -6) (Fig 2B).

### Data analytic overview

Statistical analyses were conducted using IBM SPSS Statistics Version 21.0. In no stress and stress conditions, parameter estimates were extracted from the win > fixation contrast and loss > fixation contrast using MarsBar. To create win > loss contrasts (for both stress conditions), parameter estimates from loss > fixation were subtracted from win > fixation. For our primary analyses, a *Condition* (No-Stress, Stress) x *Valence* (Win, Loss) repeated measures analysis of variance (RMANOVA) probed whether reward processing for each ROI differed as a function of stress and included eta-squared effect sizes (η^2^) where: (a) .02 - .12 = small, (b) .13 - .25 = medium, and (c) ≥ .26 = large. Significant interactions were then decomposed. A Benjamini Hochberg correction for False Discovery Rate (FDR) applied for all tests (q = .015).

## Results

### Impact of stress on reward regions

#### Left and right ventral striatum

**Fig 3:**
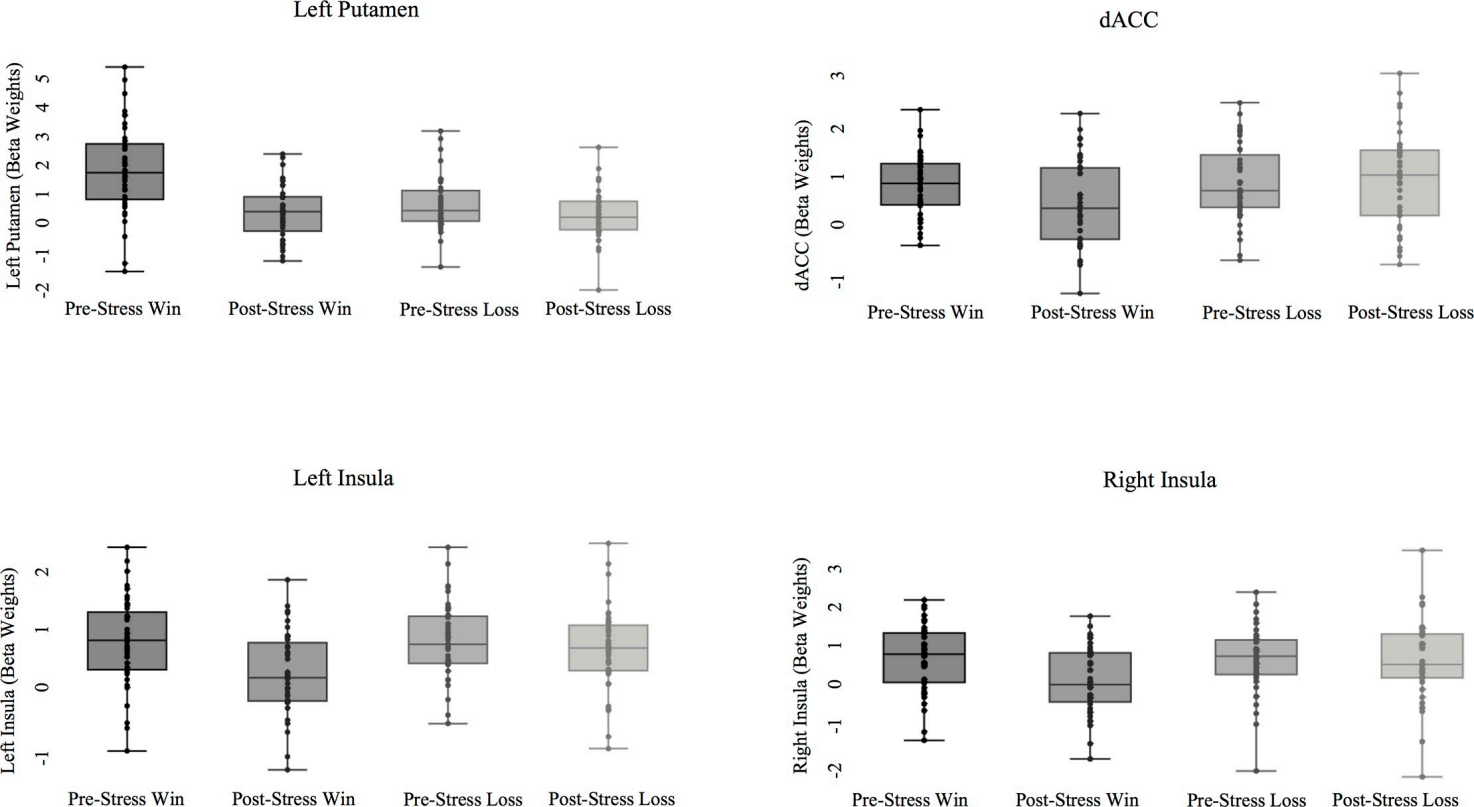
Neural activation in *no-stress* and *stress* conditions for *win* and *loss*.

The *Condition* x *Valence* interaction for the left [*F*(1,39) = 0.83, *p* = .368, *η*^*2*^ = 0.02] and right [*F*(1,39) = 3.62, *p* = .064, *η*^*2*^ = 0.09] ventral striatum were non-significant (Table 2)(Fig 3).

**Table 2:** Win- and loss-related neural activation before and after stress (*n* = 40).

	No-Stress Win *M (SD)*	No-Stress Loss *M (SD)*	Stress Win *M (SD)*	Stress Loss *M (SD)*	*F*	*p*
**Reward-Related Regions**						
Ventral Striatum						
Left	1.10 (0.68)	0.15 (0.81)	0.65 (0.74)	-0.11 (0.65)	0.83	.368
Right	0.91 (0.70)	-0.03 (0.69)	0.38 (0.85)	-0.22 (0.73)	3.62	.064
Caudate						
Left	1.17 (0.63)	0.59 (0.60)	0.84 (0.83)	0.58 (0.70)	4.47	.041
Right	1.35 (0.71)	0.68 (0.76)	1.03 (1.07)	0.65 (0.91)	2.65	.112
Putamen Left	0.66 (0.74)	0.59 (0.80)	0.48 (0.76)	0.65 (0.75)	2.22	.144
**Loss-Related Regions**						
dACC	2.02 (1.21)	2.16 (1.39)	1.23 (1.37)	2.05 (1.40)	8.29	.006
Anterior Insula						
Left	0.84 (0.68)	0.70 (0.82)	0.24 (0.68)	0.49 (0.74)	7.48	.009
Right	2.18 (1.37)	2.27 (1.29)	0.82 (1.11)	1.38 (1.16)	3.15	.084

#### Left caudate

A *Condition* x *Valence* interaction emerged for the left caudate [*F*(1,39) = 4.47, *p* = 0.041, *η*^*2*^ = 0.10]; however, this analysis does not survive our FDR correction. Nevertheless, exploratory post-hoc results show that activation following wins was greater than losses in both the no-stress (*p* = 1.20 x 10^−5^, *η*^*2*^ = 0.39) and stress condition (*p* = .008, *η*^*2*^ = 0.17). Interestingly, within-valence effects show a decrease in activation for wins following the stressor (*p* = .032, *η*^*2*^ = 0.11), but no change in response to loss (*p* = .892, *η*^*2*^ < .01) (Table 2).

#### Right caudate

The *Condition* x *Valence* interaction in the right caudate [*F*(1,39) = 2.65, *p* = .112, *η*^*2*^ = 0.06] was non-significant.

#### Left putamen

The *Condition* x *Valence* interaction in the left putamen [*F*(1,39) = 2.22, *p* = .144, *η*^*2*^ = 0.05] was non-significant.

### Effect of stress on loss regions

#### dACC

A *Condition* x *Valence* interaction emerged in the dACC [*F*(1,39) = 8.29, *p* = 0.006, *η*^*2*^ = 0.18]. Post hoc results show that activation following win was not significantly different than activation following loss (*p* = .51, *η*^*2*^ = .01) in the no-stress condition; however, activation to loss was significantly greater than win (*p* = 2.69 x 10^−4^, *η*^*2*^ = 0.30) following stress. Within-valence effects suggest that this interaction was driven by a decrease in activation to win following stress (*p* = .001, *η*^*2*^ = 0.23); contrary to our hypothesis, there was no significant increase in dACC response to loss after stress (*p* = .54, *η*^*2*^ = 0.06) (Table 2).

#### Left and right anterior insula

There was a *Condition* x *Valence* interaction in the left anterior insula [*F*(1,39) = 7.48, *p* = .009,*η*^*2*^ = 0.16]. There was no significant difference (*p* = .237, *η*^*2*^ = .04) between activation following wins relative to activation following loss in the no-stress condition. While not significant, activation for loss trended toward greater than win (*p* = .090, *η*^*2*^ = 0.17) following stress. Within-valence effects show a significant decrease in activation for win after stress (*p* = .001, *η*^*2*^ = 0.34), but not a significant increase in activation to loss (*p* = .121, *η*^*2*^ = 0.06) (Table 2). By contrast, the *Condition* x *Valence* interaction in the right anterior insula [*F*(1,39) = 3.15, *p* = .084, *η*^*2*^ = 0.08] was non-significant.

In a RMANOVA with the *win regions* (left and right Caudate, left Putamen, and left and right ventral striatum), we see a main effect of Condition (*no-stress*, *stress*), *F*(1,39) = 85.035, *p* = 0.000062, *η*^*2*^ = 0.686, with neural activity higher in both win and loss trials in the *no-stress*, relative to *stress* conditions. When we conducted comparable analyses in the *loss regions* (dACC, left and right insula), we see a main effect of Condition (*no-stress*, *stress)*, *F*(1,39) = 6.457, *p* = 0.015, *η*^*2*^ = 0.142, with neural activity higher in the *loss* trials in the *no-stress*, relative to *stress* conditions.

## Discussion

The present study tested whether social stress impacted neural activation of regions implicated in processing wins and losses in adolescents, and two principal findings emerged. First there was a blunted pattern of neural activity in the striatum in both win and loss trials during the *stress* condition compared to the *no-stress* condition. Second, in contrast to our hypothesis, acute stress did not potentiate neural responses to losses, but instead decreased neural responses to wins in loss and rejection related regions (dACC, left anterior insula). Additionally, relative to the no-stress condition, the stress condition elicited blunted neural responses to wins in the dorsal striatum, though this finding did not survive our correction for multiple tests.

Consistent with previous research in adults [23], we found blunted striatal response to reward receipt and loss during the *stress* relative to *no-stress* condition in adolescents. This change in reward receipt and loss in response to stress may be indicative of a stress-elicited anhedonic-like pattern of neural activation. More broadly, this suggests that the experience of stress, particularly social stress, may cause diminished neural responsivity in reward-related regions, which may be a factor for diminished positive affect that often characterizes adolescent depression [43].

In regions that have been implicated with responses to losses (i.e., dACC, anterior insula), no differences emerged in neural activation following losses and wins in the no-stress condition. In contrast, following stress, we observed a greater response to loss relative to win conditions. A closer examination revealed that following stress, this effect was driven by a blunted response to wins as opposed to greater sensitivity to loss. This finding suggests that this potential vulnerability may not be a byproduct of loss sensitivity, but rather, a consequence of reduced responsiveness to positive experiences, which is broadly consistent with prior work implicating disruption of positive affective processes in stress-related disorders [43]. Moreover, this finding suggests that diminished responsivity to rewards may not be restricted to the typical neural circuitry of reward (*e*.*g*., striatum), but also may affect the responsivity within frontal regions in adolescents.

Though exploratory, acute stress contributed to blunted patterns of activation in the left dorsal striatum, a region implicated in reward-related learning [44], which is in line with prior stress exposure findings probing striatal activation in the context of stress exposure [22]. Prior research suggests that caudate activation is strongest to unpredictable rewards that are contingent on an individual’s actions [45]. Our findings of the left dorsal striatum—despite not surviving the Type I correction—may indicate a decrease in the reinforcement of goal-directed actions following stress. This interpretation would be consistent with prior work demonstrating a weaker caudate response to reward receipt in individuals with stress-related disorders (i.e., major depressive disorder) relative to healthy controls [4].

This study builds on previous investigations of stress and reward-related dysfunction in two ways. First, this study expands previous findings in adults [23] to adolescents. Prior research with adults shows that acute stressors result in decreased sensitivity to reward in reward-related regions (e.g., dorsal striatum, ventral striatum)[22, 23]. Given the heightened saliency of reward during adolescence, significant social stressors, and increased rates of psychiatric disorders, testing the relationship between reward and interpersonal stress in adolescence is critical for understanding the development of psychopathology. Additionally, in contrast to previous work testing early life adversity or chronic stress, the pre-post study design allows us to identify changes in neural response as a direct result of interpersonal stress. Our findings provide a model by which stress may expose a vulnerability to reward-related dysfunction, thus increasing risk for psychopathology. These findings are consistent with studies demonstrating that an increase in cortisol, a marker of stress, is associated with a blunted reward-related response in healthy adults [46].

Prior research suggests that adolescents’ make poorer decisions when exposed to social or cognitive stressors. This impaired decision-making appears to be an exaggeration of their individual risk-taking profiles under no-stress conditions, thus suggesting both an individual differences and environmental effect on decision-making in adolescents [47]. Our findings extend this literature by suggesting that in adolescents, there may be changes in reward- and loss-responsivity under stress.

Findings should be interpreted in light of a few limitations. First, the ethnic distribution within this sample limits the generalizability of the findings. Additionally, the age range was restricted to 12 to 14 years old in order to reduce putative age-related effects. However, this restricted range may also limit the generalizability of these findings to younger or older youth. Second, the Guessing Task uses a fixed random sequence design, which prohibits evaluating reinforcement learning in response to reward feedback. Thus, in this study, changes in behavioral response to stimuli (e.g., response time) were not collected. However, these data could have provided additional information in regard to behavioral changes pre- and post-stress. Lewis and colleagues [48] found that when engaged in a learning condition paradigm, adults under physical stress showed a blunted response in the striatum in response to monetary gains, relative to adults in a no-stress condition. Given that these findings are in contrast to our own, it would be important to investigate learning conditions in a similar paradigm with adolescents, as there may be developmentally differences in neural response to rewards under stress during learning. Additionally, this study would have benefitted from a physiological measure of stress responsivity rather than relying on self-reported changes in affect alone. Finally, the study design required the use of the Guessing Task in both the no-stress and stress conditions, leaving open the possibility of task habituation. Randomization of the task conditions was not possible in this particular design. While the repetition of this task was a critical part of our study design, it is possible that there could be a general dampening of response to the second presentation of the stimuli. Without the counterbalance it is impossible for us to know whether there were specific carryover effects.

### Summary

In sum, the current findings identify how neural response to reward may change following social stress in adolescents. Building on prior work in experimental animals [12–15] and human adults [23], we illustrate the importance of conceptualizing adolescent neural vulnerability within the presence of stress, as this may clarify risk for mental disorders during a critical developmental period.

## Supporting information

S1 Reward Data Reward Data. The reward data file contains all data included in the analyses for this paper.
(SAV)
